# The Quarantine Conference

**Published:** 1889-04

**Authors:** 


### THE QUARANTINE CONFERENCE.

The conclusions reached by the Quarantine Conference, in session at Montgomery, March 5, 6, and 7, were of some importance and value, and will no doubt interest our readers.

The Conference was composed of distinguished physicians and sanitarians of the Southern States, and representatives of the United States Army, Navy and Marine Hospital service.

The method of disinfection as practiced at the New Orleans station, by the use of super-heated steam, in steel cylinders under pressure, was endorsed as being the best method now known to science.

The Surgeon-General of the Marine Hospital service (who was present) was requested and promised to erect at Tampa, Fla., a similar plant. The administration of marine quarantine, as now carried out by the Surgeon-General, was especially commended, and the request was made that more stations and more men be devoted by him to this work.

The co-operation of the management of the Plant Line of steamers, plying between Havana and Tampa, with Dr. Burgess, U. S. Medical Inspector at Havana, was commended as an example for cleanliness of ships, scrutiny of passengers and disinfection of baggage.

By special resolution, the attention of the Secretary of the Treasury of the United States was called to the prevalence of smuggling between Cuba and the Florida coast, and the great danger of introduction of yellow fever by this illicit traffic, and he was requested to use additional precautions and, if possible, put a stop to it.

On the question of inland quarantine it was decided that, as far as possible, this should always be declared, where they exist, by State Boards of Health, and that by whomsoever declared, within thirty-six hours after the proclamation, comfortable quarters, with provisions and bedding, must be provided for the unfortunates detained at the station.

The Conference, by a decided vote, refused to endorse the proposition that it was necessary to disinfect a town or city, in which yellow fever had prevailed, but in which there had been no cases for several months, and the place had been subjected to the frosts and freezes of winter; deeming that the use of disinfectants under these circumstances, was not only useless but tended to breed unnecessary terror and distrust, not only among the people of the place, but of surrounding States.

